# Case Report: Stroke-like migraine attacks after radiation therapy syndrome: a rare complication 26 years after cranial radiotherapy

**DOI:** 10.3389/fonc.2023.1202918

**Published:** 2023-10-02

**Authors:** Olga Duranikova, Igor Straka, Lubomir Melichercik, Peter Marcek, Karin Gmitterova, Peter Valkovic

**Affiliations:** ^1^ 2nd Department of Neurology, Faculty of Medicine, Comenius University in Bratislava, University Hospital Bratislava, Bratislava, Slovakia; ^2^ Department of Magnetic Resonance Imaging, Dr. Magnet Ltd., Bratislava, Slovakia; ^3^ Institute of Normal and Pathological Physiology, Centre of Experimental Medicine, Slovak Academy of Sciences, Bratislava, Slovakia

**Keywords:** stroke-like migraine attacks after radiation therapy syndrome, SMART syndrome, brain tumor, cranial radiotherapy, neurooncology

## Abstract

Stroke-like migraine attacks after radiation therapy (SMART) syndrome is a rare delayed complication of cranial radiotherapy, that may present decades after brain irradiation. Here we present a case of 41-year old patient with a history of grade 3 oligodendroglioma, epilepsy and migraine, 26 years after brain radiation therapy, who was admitted with right hemicranial headache, nausea, left homonymous hemianopsia, weakness of the left arm and left-sided hemihypesthesia. After considering alternate diagnoses, we ultimately diagnosed SMART syndrome. Despite its rare occurrence and unknown pathophysiology, there are more case reports of SMART syndrome reported due to advancements in oncology treatment and increasing patients’ survival rates. Therefore, diagnosis of SMART syndrome should always be considered in patients with a history of cranial radiation presenting with focal neurologic deficits and migraine, especially with a change in pattern of their usual migraine attack.

## 1 Introduction

Stroke-like migraine attacks after radiation therapy (SMART) syndrome is a rare delayed complication of cranial radiotherapy characterized by a combination of clinical and radiological findings that may present decades after brain irradiation. It is defined by episodes of recurrent headaches associated with seizures and focal neurological deficits with just about 100 case reports in the literature (1). Exact pathophysiology is still unknown, although some authors suggest endothelial damage leading to impaired autoregulation, radiation vasculopathy or cerebral hyperexcitability leading to seizures and headache (2). Typical magnetic resonance imaging (MRI) patterns show transient unilateral cortical gadolinium enhancement and an increased T2 signal with cortical thickening predominantly in temporal, parietal and occipital cortex (3). Although considered as a diagnosis of exclusion, there are more case reports of SMART syndrome reported due to advancements in oncology treatment and increasing patients’ survival rates. We present a case of a patient presenting with SMART syndrome nearly 30 years after cranial radiation with a complete resolution of symptoms after corticosteroid therapy.

## 2 Case report

We present a case of a 41-year old male patient with a history of grade 3 oligodendroglioma (1p/19q testing and IDH testing were unknown) in the right frontal lobe, which was treated with gross total resection (1995), adjuvant chemotherapy with procarbazine and vincristine (1996) and cranial radiation therapy (60 Gray/30 fractions) in 1996-1997. He received high-dose radiotherapy for the whole right hemisphere (in that period localized radiotherapy was not possible). According to his medical history, the patient had secondary epilepsy treated with valproate and had been without seizures for several years. He also suffered from episodic migraines with aura since 2014 that were treated with over-the-counter analgesics. The patient was treated for arterial hypertension with metoprolol as well. Other medical history was irrelevant.

**Figure 1:**
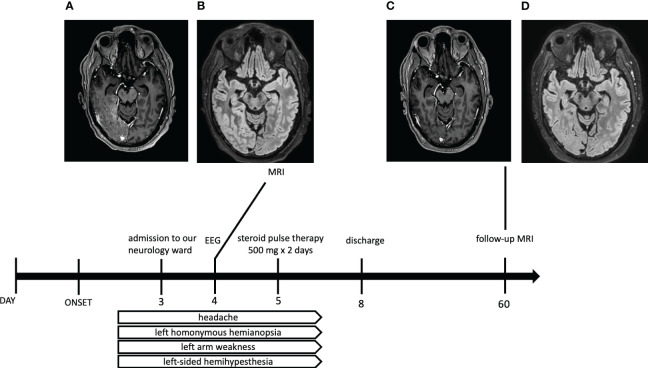
Timetable of clinical and radiological course of our case report of SMART syndrome. Brain MRI scans (**(A)** – postcontrast T1-weighted, **(B)** – FLAIR sequence) one day after admission to the hospital showing swelling and hyperintensities of right temporal, parietal and occipital cortex on FLAIR (fluid-attenuated inversion recovery) with corresponding cortical and leptomeningeal enhancement on the postcontrast T1-weighted image. Corresponding MRI two months after discharge (**(C)** – postcontrast T1-weighted, **(D)** – FLAIR sequence) showing complete resolution of cortical enhancement.

At admission to our neurology ward (March 2022) he presented with right hemicranial headache and nausea, loss of visual field on the left, left-sided paresthesias and weakness that started 3 days prior to admission. It had different characteristics than his usual migraine attacks – his aura typically consists of paresthesias of the left upper limb with resolution in few minutes, followed by unilateral pulsating headache with nausea, vomitus, photophobia and phonophobia. Neurology examination revealed left homonymous hemianopsia, mild weakness of the left arm and left-sided hemihypesthesia. Laboratory results including complete blood count, biochemical, and immunological analyses for humoral and cell-mediated immunity were normal. Initial brain CT scan showed post-radiation porencephaly and gliosis with no sign of new neoplasm or stroke. His electroencephalogram showed no epileptiform activity. He underwent brain MRI that revealed swelling and hyperintensity of right parietal, temporal and occipital cortex on FLAIR (fluid-attenuated inversion recovery) with corresponding cortical and leptomeningeal enhancement on the postcontrast T1-weighted image. In order to exclude neuroinfection, we planned to do a lumbar puncture, which was not performed due to absence of patient’s consent. We diagnosed SMART syndrome (stroke-like migraine attacks after radiation therapy) in correlation with the patient’s previous history of radiation therapy of central nervous system, clinical features and MRI findings. Intravenous methylprednisolone (1000 mg in 2 days) was administered with subsequent complete resolution of the patient’s symptoms. Because of patient’s chronic use of beta blockers for arterial hypertension, we could not administer verapamil due to the risk of severe bradycardia. We added candesartan as prophylactic medication for migraine with a significant reduction in migraine days. A follow-up MRI was obtained two months later showing complete resolution of cortical enhancement, which is consistent with the diagnosis of SMART syndrome (**Figure 1**). The patients is examined at our outpatient clinic every six months with last appointment in June 2023, he has not had any relapses of the initial symptoms since discharge from the hospital.

## 3 Discussion

We presented a case report of 41-year old patient with a history of grade 3 oligodendroglioma, 26 years after cranial radiation therapy, which fulfilled clinical and radiological criteria for SMART syndrome. SMART syndrome is a very rare late complication of brain radiation that can manifest 1 to 37 years after brain radiation with an average of 9.5 years reported by Black et al. (4, 5). Radiation dose associated with SMART syndrome is usually more than 50 Gray, but there have also been lower doses reported in the literature (6). Although the first described case of SMART syndrome dates back to 1995, its pathophysiology is still not well understood. There are few proposed hypotheses mentioned in the literature. The first one suggests a delayed effect of cranial radiation on cerebral vessels leading to impaired autoregulation and disruption of the blood-brain barrier, hyperperfusion and neurologic dysfunction and enhancement on MRI. This mechanism also resembles posterior reversible encephalopathy syndrome (PRES) predominantly affecting the parieto-occipital area, which is more prone to radiation damage. The second proposed mechanism involves trigeminovascular system activation that ultimately decreases the threshold to cortical spreading depression, which is characteristic in pathophysiology of migraine (7, 8). Some authors also suggest that SMART syndrome could be an expression of migraine or seizures that are not related to previous radiation (9). Seizures have been described as a common accompanying symptom in SMART syndrome and in larger case series seizures were present in 35% and 64% of patients. Because of its high prevalence and resemblance of MRI findings with postictal changes led some authors to think, whether parieto-occipital cortical damage is a result of radiation and is therefore prone to seizures. Another hypothesis is based on the fact, that recurrent seizures are responsible for cortical abnormalities (7). On the contrary, Singh et al. revealed that half of the patients in their study diagnosed with SMART syndrome did not have epileptiform discharges and resolution of EEG changes were not consistent with resolution of symptoms (2). Symptoms of SMART syndrome typically consist of recurrent attacks of headache, seizures and focal neurological deficits (aphasia, hemianopsia, hemiparesis, sensory disturbances, negligence); sometimes cognitive impairment is present. Because of its rare occurrence and clinical picture resembling stroke, it may manifest as a diagnostic dilemma in clinical practice. Symptoms are usually transient and fully reversible, although incomplete resolution of symptoms as well as neurologic sequelae have been described in some cases as well (10, 11).

Diagnosis is based on revised Black et al. criteria, which include a remote history of external cranial irradiation without evidence of residual or recurrent neoplasm, focal neurological sign referable to a unilateral cortical region and cortical enhancement on MRI within the field of previous irradiation (**Table 1**) (3). Other characteristic MRI patterns include cortical thickening with increased T2/FLAIR signal. Diffusion-weighted images show T2 shine through effect predominantly, while diffusion restriction is usually minor. SMART syndrome should be a diagnosis of exclusion. In differential diagnosis we have to consider mitochondrial encephalomyopathy lactic acidosis and stroke-like episodes (MELAS), status epilepticus with Todd’s paresis. Other conditions that can mimic SMART syndrome include tumor recurrence, familial or sporadic hemiplegic migraine, PRES, cerebral vasculitis, cerebral amyloid-associated inflammation, infective or immune-mediated meningoencephalitis, acute late-onset encephalopathy after radiotherapy and focal cerebral radiation necrosis (11, 13). Given the small number of cases with SMART syndrome, there is no clear consensus regarding treatment. Several reports suggest using corticosteroids, although the dose is in many cases not specified (14). For long-term prophylaxis of SMART syndrome, verapamil was used successfully in some cases (11).

**Table 1:** Black et al. revised diagnostic criteria for Stroke-like migraine attacks after cranial radiation therapy syndrome (3, 12).

A. Remote history of external beam cranial irradiation without evidence of residual or recurrent neoplasm
B. Prolonged, reversible signs and symptoms referable to a unilateral cortical region beginning years after irradiation Manifestations may include: • confusion, • seizures, • headache with the attacks, • visuospatial deficits, • hemisensory deficits, • hemiparesis, • aphasia, • antecedent migraine with or without aura starting after irradiation
C. Transient, diffuse, unilateral cortical gadolinium enhancement of the cerebral gyri sparing the white matter within a previous radiation field
D. Not attributable to another disorder

## 4 Conclusion

We presented a case of SMART syndrome as a late complication of radiotherapy in a patient with a history of grade 3 oligodendroglioma. It is a diagnosis of exclusion, but it should always be considered in patients with a history of cranial radiation presenting with focal neurologic deficits and migraine, especially with a change in pattern of their usual migraine attack. Despite its rare occurrence there are still more reported cases of SMART syndrome with advancement in oncology strategies and better survival rates.

## Data availability statement

The raw data supporting the conclusions of this article will be made available by the authors, without undue reservation.

## Ethics statement

Written informed consent was obtained from the participant/patient for the publication of this case report.

## Author contributions

OD, IS, LM, PM, KG, and PV obtained and analyzed the clinical data. OD wrote the manuscript. PV and IS designed and supervised the study. IS, LM, PM, KG, and PV reviewed and edited the manuscript. All authors contributed to the article and approved the submitted version.
